# Fatigue Is Common in Immunoglobulin G Subclass Deficiency and Correlates With Inflammatory Response and Need for Immunoglobulin Replacement Therapy

**DOI:** 10.3389/fimmu.2021.797336

**Published:** 2022-01-10

**Authors:** Per Wågström, Åsa Nilsdotter-Augustinsson, Mats Nilsson, Janne Björkander, Charlotte Dahle, Sofia Nyström

**Affiliations:** ^1^ Department of Infectious Diseases, Ryhov County Hospital, Jönköping, Sweden; ^2^ Department of Biomedical and Clinical Sciences, Linköping University, Linköping, Sweden; ^3^ Division of Inflammation and Infection, Department of Biomedical and Clinical Sciences, Linköping University, Linköping, Sweden; ^4^ Department of Infectious Diseases, The Vrinnevi Hospital, Norrköping, Sweden; ^5^ Futurum, Academy of Health and Care, Region Jönköping County, Jönköping, Sweden; ^6^ Wetterhälsan, Health Care Centre, Jönköping, Sweden; ^7^ Division of Clinical Immunology and Transfusion Medicine, Department of Biomedical and Clinical Sciences, Linköping University, Linköping, Sweden; ^8^ Division of Molecular Medicine and Virology, Department of Biomedical and Clinical Sciences, Linköping University, Linköping, Sweden

**Keywords:** primary antibody deficiency, immunoglobulin G subclass deficiency, fatigue, inflammation, cytokine, IgRT, quality of life

## Abstract

**Purpose:**

Individuals with immunoglobulin G deficiency (IgGsd) often complain of fatigue. The correlation between systemic inflammation and fatigue is unknown. In this study perceived quality of life (QoL) and fatigue in individuals with IgGsd, on and off immunoglobulin replacement therapy (IgRT) were correlated to inflammatory markers in plasma to identify the subgroup that benefits from IgRT.

**Method:**

Thirty-five IgGsd-patients were sampled on three occasions: at baseline, after being on IgRT for at least 18 months, and 18 months after discontinuation of IgRT. Short form 36, EQ-5D-5L visual analogue scale and fatigue impact scale questionnaires were used for evaluation of QoL and fatigue. Furthermore, a panel of 92 inflammatory markers were analysed in plasma. Thirty-two gender- and age-matched healthy individuals were included as controls and sampled on one occasion.

**Results:**

QoL was lower and perceived fatigue higher in IgGsd compared to the controls. Severe fatigue and low QoL were associated with the need to restart IgRT (which is considered in IgGsd-individuals with a high burden of infections in Sweden). Twenty-five inflammatory factors were dysregulated in IgGsd and the plasma protein patterns were similar regardless of whether IgRT was ongoing or not. Enrichment analysis indicated IL-10 signalling as the most affected pathway. Severe fatigue was associated with decreased levels of the neurotrophic factors VEGFA and CSF-1.

**Conclusion:**

Fatigue is a major contributory factor to impaired health-related QoL in IgGsd and is related to the need for IgRT. Low-grade systemic inflammation is a potential driver of fatigue. In addition to the burden of infections, we suggest the degree of fatigue should be considered when the decision to introduce IgRT is made.

## Introduction

Health-related quality of life (QoL) is a multidimensional concept that relate to an individual’s perceived physical and mental health. Fatigue is a major cause of reduced QoL in many diseases, including cancer and chronic inflammatory disorders. In these conditions, fatigue has been reported to be associated with a poor prognosis and may even predict mortality (1–4). Primary antibody deficiencies (PADs) comprise a group of rare disorders characterised by an inability to produce effective antibody responses. PADs are associated with increased susceptibility to infections and in some cases with chronic inflammation. Common variable immunodeficiency (CVID) is a PAD, characterised by reduced levels of total IgG and standard care includes lifelong immunoglobulin replacement therapy (IgRT) (5). IgG subclass deficiency (IgGsd) is a more common type of PAD and usually entails a less severe clinical course than CVID. IgGsd is characterised by reduced levels of IgG1, IgG2 or/and IgG3 (6, 7). The clinical presentation of IgGsd ranges from lack of any symptoms to recurrent respiratory tract infections that can result in chronic lung damage (8, 9). In Sweden, the need for IgRT in IgGsd is based on the frequency and severity of bacterial infections and/or the presence of lung damage. According to national guidelines in Sweden, all patients with IgGsd who receive IgRT should undergo a discontinuation trial after 18 months of treatment. If bacterial respiratory tract infections reoccur, or if signs of reduced lung function develop, IgRT should be restarted (5).

Low QoL has been reported in PADs and often affects both physical and mental health (10). Fatigue is overrepresented in PADs, with the highest prevalence among individuals with CVID. Furthermore, depression is the most common comorbidity in CVID with fatigue (11, 12). The prevalence of fatigue among individuals with IgGsd is unknown and studies on health-related QoL of those with this condition are lacking.

The underlying mechanisms of fatigue are multifactorial and poorly understood. Several studies suggest that pro-inflammatory cytokine signalling, as part of the inflammatory reaction, is of importance (13–16). In healthy individuals, an injection of low-dose recombinant interleukin (IL)-6 has been found to be associated with increased fatigue (17). Interferon therapy in patients with hepatitis C and multiple sclerosis is known to increase the risk of developing depression (18, 19). Dysregulation of inflammatory factors in plasma has also been reported in chronic fatigue syndrome/myalgic encephalitis (CFS/ME), a condition characterised by severe fatigue and signs of immune dysregulation (20, 21). Together, these findings suggest that inflammation is associated with mood disturbances. However, though many of the new anti-inflammatory treatment strategies, *i.e.* biologicals, effectively control the inflammatory process, they do not consistently reduce fatigue (22). Thus, other mechanisms are also important drivers of fatigue and low mood in diseases characterised by chronic inflammation.

In this longitudinal prospective study, the general QoL and prevalence of fatigue in a Swedish cohort of IgGsd patients, were assessed during and after discontinuation of IgRT. In addition, systemic inflammation was evaluated by plasma profiling of inflammatory mediators while on, and off IgRT. We found that low QoL and fatigue were common in IgGsd regardless of whether IgRT was ongoing or not, and were most pronounced in those who needed to reintroduce IgRT due to the recurrence of frequent infections. The plasma protein profiles in IgGsd reflected increased IL-10 signalling and were not convincingly affected by IgRT. In addition, IgGsd fatigue correlated negatively with the plasma levels of macrophage colony stimulating factor 1 (CSF-1). Several other inflammation-related growth factors were also decreased in IgGsd complicated by severe fatigue.

## Methods

### Ethics

The study subjects signed informed consent forms and the study was approved by The Ethical Review Board in Linköping, Sweden (Dnr 2011/506-31). It follows the World Medical Association Declaration of Helsinki on ethical principles for medical research on humans (23).

### Study Subjects

**Figure 1:**
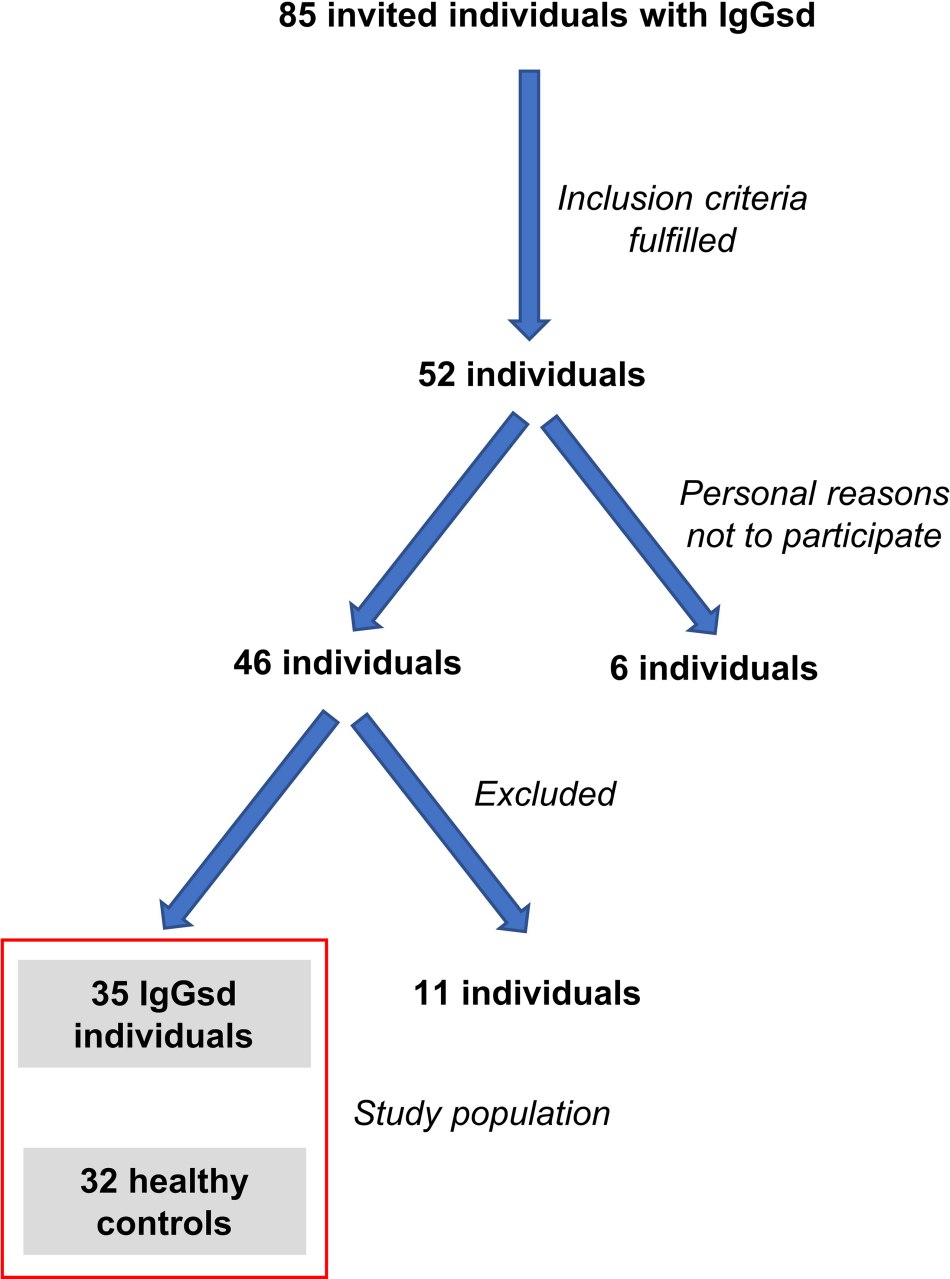
Flow chart of individuals included in the study. Of 85 individuals with IgG subclass deficiency (IgGsd) 52 met the inclusion criteria of the study. Six individuals chose not to participate due to personal reasons. Another 10 individuals were excluded since they did not start IgG-replacement therapy in time for inclusion and one individual was excluded due to unconfirmed diagnosis. Healthy controls were recruited among blood donors and matched for gender and age.

Participants with IgGsd were recruited from and followed up by the Departments of Infectious Diseases in the counties of Jönköping and Östergötland in Sweden. All were 18 years of age or above, with a confirmed IgGsd diagnosis. No one had a known serious lung disease or had previously stopped treatment on a trial basis. Eighty-five patients with IgGsd were invited to participate. Eighteen were excluded due to known severe lung disease, since discontinuation of IgRT is not advisable in these cases. Another fifteen were excluded from participating since they had previously experienced a period of discontinuation of IgRT. Out of the 52 individuals who met the inclusion criteria, six chose not to participate for personal reasons, 10 patients did not start the treatment in time to be included in the study and one individual was later excluded due to unconfirmed IgGsd diagnosis (**Figure 1**). In total, 35 patients were included (21 women and 14 men) out of which 12 (seven women and five men) were newly diagnosed and had not yet started IgRT at baseline, **Supplementary Information (SI)** **Figure 1**. The other 23 (12 women and 11 men) were already on IgRT and had been for periods ranging from two months up to several years. Blood samples were collected, and QoL questionnaires were answered on three occasions in the patient group: 1) at baseline, 2) after being on IgRT for at least 18 months and 3) 18 months after discontinuation of IgRT, or earlier if the IgRT needed to be restarted due to recurrence of frequent infections. Thirty-two healthy blood donors matched for gender (13 men and 19 women) and age (median 54, range 28-68 years), served as controls. Controls were blood-sampled once, and 24 completed the QoL questionnaires on one occasion (**SI Figure 1**).

### Short Form-36 and EuroQoL-5 Dimension-5 Level Visual Analogue Scale

The 36-item short-form (SF -36) and the Euro QoL-5 Dimension-5 Level visual analogue scale (EQ-5D-5L VAS) generic questionnaires were used to measure health-related QoL. SF-36 measures 36 items across eight different dimensions: physical functioning, role limitations due to physical problems, bodily pain, general health, vitality, social functioning, role limitations because of emotional problems and mental health. The SF-36 vitality dimension specifically reflects fatigue (24). The scores are weighted sums of the questions in each dimension, transformed to a 0–100 scale (25). A low SF-36 score correlates to poor QoL. The EQ-5D-5L VAS is a tool measuring overall health where the rater selects a number between one and 100 to describe the condition of their health, 100 being the best health imaginable.

### Fatigue Impact Scale

The prevalence of fatigue and its impact on everyday life was assessed by the Fatigue Impact Scale (FIS), which is one of the most widely used tools for specifically assessing chronic fatigue (26). The FIS consists of 40 items that are scored 0 (no problem) to 4 (extreme problem), generating a continuous scale of 0-160. The items cover three areas that describe how fatigue impacts physical (10 items), cognitive (10 items) and psychosocial (20 items) functionality. A high FIS score correlates to more severe fatigue.

### Profiling of Inflammatory Proteins in Plasma

After centrifugation of blood samples, plasma was collected and stored at -80°C prior to analysis. Frozen samples were sent to the Clinical Biomarkers facility, Science for Life Laboratory, Uppsala University (Uppsala, Sweden) where they were analysed following standard operating procedures. The multiplex protein extension assay (PEA) inflammation panel (Olink Target Inflammation v.95302) was used for detection of 92 different inflammatory associated proteins (Olink Proteomics, Uppsala, Sweden) (27, 28).

### Statistical Analyses

For comparisons between the groups, the Wilcoxon test was used for ordered categorical data and the Student’s t-test was used for continuous variables. Multivariate statistical analysis of plasma inflammatory factors was carried out using a two-tailed t-test in Microsoft Excel. The *p* value for each factor was adjusted for multiple comparisons using the False Discovery Rate (FDR) by the Benjamini–Hochberg procedure. The Reactome version 76, gene list tool was used to explore the signalling pathways enriched in differently expressed inflammatory factors (29). Factors that significantly differed between groups and that were upregulated >1.1 times or down-regulated <0.9 times were used in enrichment analysis. Statistical significance was defined as p<0.05. SAS/STAT^®^ ver. 13.1 software, (Copyright ^©^ 2002-2012 by SAS Institute Inc., Cary, NC, USA), Statistica Ver.13, (Copyright ^©^ 1984-2017, TIBCO Software Inc., CA, USA) and Graphpad Ver. 8 (^©^ 2020, Graphpad Software., CA, USA) were used for the calculations. Graphpad was also used for graphics.

## Results

### The Changes in the Study Population During the Course of the Study

At baseline, 12 study subjects were treatment-naïve while 23 were already on IgRT. At month 18, 34 subjects were sampled and answered the QoL questionnaires. At this point, one had already left the study due to comorbidity. At month 36, after 18 months of discontinuation of IgRT, only 25 subjects had been sampled and answered the QoL questionnaires, because six had started IgRT without reporting QoL, one had left the study and two had died (unrelated to IgGsd). Out of these remaining 25 subjects, 12 (48%) needed to restart IgRT while 13 did not. In total, out of the 35 subjects that were recruited to the study, 18 (51%) needed to restart IgRT after the period of discontinuation, while 16 did not need the substitution and one had left the study.

### Demographics, Comorbidities, and Ig Replacement Therapy

There were no differences in age between the group of the IgGsd individuals who needed to restart IgRT (median 54, range 32-72 years) and those who did well without IgRT (median 55, range 32-78 years). There were more women than men included in the study (**Table 1**), and the distributions of men and women were similar among individuals who needed IgRT and among those that did well without IgRT (**SI Table 1**). The group of IgGsd individuals was heterogeneous, with different types of comorbidities (**Table 1**). At inclusion, two IgGsd individuals had ongoing anti-depressive treatment and another four had a history of depression. Sixteen (46%) individuals had a chronic lung disease and 13 of them had been diagnosed with asthma, two with COPD, and one with emphysema. Seventeen (49%) of the IgGsd individuals also had at least one autoimmune disease (**Table 1**) and diabetes was the most common (17%). Two IgGsd individuals (6%) had been diagnosed with inflammatory bowel disease, another two with goitre and Sjögren’s syndrome, respectively. Other diagnoses of autoimmunity included haemolytic anaemia, vitiligo, iritis, psoriasis and Churg-Strauss syndrome. Atopy was present in eleven (31%) individuals. Out of 16 individuals with lung disease 11 needed to restart IgRT including the individuals with COPD and emphysema (**SI Table 1**). Nine (52%) of the patients with autoimmune disease needed to restart IgRT. Overall, the presence of comorbidities did not have any major impact on the need for IgRT.

**Table 1:** Demographics and comorbidity of study participants with IgG subclass deficiency.

Gender, *n* women/men (%)	21/14	(60/40)
Age, median years (range)	58	(20–72)
Healthcare/service profession, *n* (%)	11	(31)
Depression, *n* (%)	2	(6)
Low lung function, *n* (%)	5	(14)
Lung disease, *n* (%)	16	(46)
Bronchiectasis, *n* (%)	12	(34)
Ever smoker, *n* (%)	8	(23)
Autoimmunity, *n* (%)	17	(49)
Atopy, *n* (%)	11	(31)
Low total IgG, *n* (%)*	18	(51)
Specific antibody deficiency, *n* (%)^*^#^^	6	(17
IgG1 deficiency, *n* (%)*	14	(40)
IgG2 deficiency, *n* (%)*	13	(37)
IgG3 deficiency, *n* (%)*	19	(54)
IgGsd mixed deficiency, *n* (%)^*^	8	(23)
Low IgA^*^§^^	Sep-00	(26)

^*^Without Ig replacement therapy, ^#^deficiency of pneumococcal antibodies and haemophilus influenzae serotype B antibodies, ^§^none of the participants had IgA<0.07 g/L.

The control population was in good physical and mental health. Three women and one man (all 62 years or older) reported regular use of antihypertensive drugs. One of the controls was on amitriptyline for neuropathic pain, no other antidepressant treatment was reported by the controls. Six of the controls reported occasional intake of non-steroidal anti-inflammatory drugs and/or paracetamol during the week before blood sampling.

### Poor Self-Rated Health and the Need for IgG Replacement Therapy

**Figure 2:**
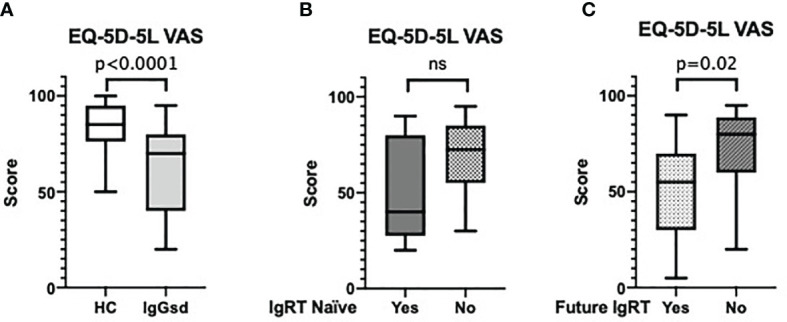
IgGsd self-reported QoL by the EQ-5D-5L VAS. IgGsd individuals reported poorer overall QoL by EQ-5D-5L VAS than healthy controls **(A)**. At baseline there were no differences in EQ-5D-5L VAS scores between treatment naïve IgGsd individuals (n=12) and individuals (n=23) already on IgRT **(B)**. After 18 months of IgRT discontinuation, at time-point 36 months EQ-5D-5L VAS scores were poorer in IgGsd individuals (n=12) that needed to reintroduce IgRT compared to individuals (n=13) who did not **(C)**. VAS, visual analogue scale; QoL, quality of life; IgGsd, immunoglobulin subclass deficiency; IgRT, immunoglobulin replacement therapy; ns, not significant.

At baseline, SF-36 revealed significantly poorer health-related QoL in all eight SF-36 dimensions, regardless of whether the subjects were already on IgRT or treatment-naïve, compared to healthy controls (**Table 2A**). Lower QoL was also reported at baseline by the EQ-5D-5L VAS (**Figure 2A**) without any differences between the treatment-naïve individuals and those already on IgRT (**Figure 2B**). Individuals who later needed to restart IgRT reported lower scores in most SF-36 dimensions, physical functioning being an exception, both at baseline and 18 months after IgRT discontinuation (time point 36 months), compared to those who performed well without IgRT (**Table 2A**). They also reported poorer EQ-5D-5L VAS scores compared to those who did not need to restart IgRT (**Figure 2C**). Taken together, compared to healthy controls self-rated QoL was decreased in IgGsd, and most pronounced in individuals in need of IgRT.

**Table 2A:** SF-36 health-related quality of life in IgGsd and controls at different time points.

Study subjects	N		PF	RP	BP	GH	VT	SF	RE	MH
Baseline										
IgGsd	35	Median	80	69	51	30	35	63	92	60
		25th-75th percentile	60-95	44-94	32-84	20-42	15-50	38-100	67-100	40-72
Controls	24	Median	95	100	84	92	60	100	100	72
		25th-75th percentile	90-100	94-100	72-100	77-97	50-70	88-100	100-100	68-76
			**p<0.01**	**p<0.001**	**p<0.01**	**p<0.0001**	**p<0.0001**	**p<0.001**	**p<0.001**	**p<0.01**
Need IgRT	18	Median	70	56	41	20	20	50	83	56
		25th-75th percentile	50-90	25-75	31-74	10-45	10-35	38-100	50-100	32-68
No need IgRT	17	Median	85	84	51	35	43	88	96	66
		25th-75th percentile	68-98	69-97	41-100	25-45	35-58	63-100	79-100	56-72
			**NS**	**p<0.02**	**NS**	**p<0.05**	**p<0.01**	**p<0.01**	**NS**	**p=0.05**
18 months										
Need IgRT	18	Median	88	63	47	42	20	63	79	50
		25th-75th percentile	55-95	44-75	41-74	15-45	20-40	50-75	50-100	36-72
No need IgRT	16	Median	85	72	57	32	40	81	83	62
		25th-75th percentile	65-95	63-88	41-79	25-56	33-60	56-100	71-100	46-72
			**NS**	**NS**	**NS**	**NS**	**p<0.05**	**p<0.05**	**NS**	**NS**
36 months										
Need IgRT	12	Median	75	44	41	25	18	44	50	32
		25th-75th percentile	50-90	22-66	31-51	15-37	3-28	19-75	46-79	26-56
No need IgRT	13	Median	80	84	68	53	43	94	96	66
		25th-75th percentile	65-95	63-100	51-100	30-67	30-60	75-100	67-100	48-72
			**NS**	**p<0.01**	**p<0.05**	**p<0.01**	**p<0.01**	**p<0.01**	**p<0.05**	**p<0.01**

PF, physical functioning; RP, role limitations due to physical problems; BP, bodily pain; GH, general health; VT, vitality; SF, social functioning; RE, role limitations because of emotional problems; MH, mental health; IgGsd, IgG subclass deficiency; IgRT, immunoglobulin replacement therapy; NS, not significant.

Values in bold indicate statistically significant.

### Chronic Fatigue Is Common in IgG Subclass Deficiency

Fatigue is closely related to the SF-36 dimension of vitality. At all time-points of assessment, the IgGsd individuals that performed well without IgRT after discontinuation scored better on SF-36 vitality than individuals that needed to restart IgRT, but still poorer than the controls (**Table 2A**). Based on the SF-36 vitality score, fatigue is common in IgGsd and is most pronounced in individuals in need of IgRT (**Table 2A**).

We also investigated the prevalence of fatigue using the FIS questionnaire which specifically assessed chronic fatigue. At baseline there was a significantly poorer total fatigue score in the whole IgGsd group compared to controls, with poorer scores in all three areas of FIS (**Table 2B**). Sixteen out of 35 subjects reported FIS total scores ranging between 60 and 138, compared to one (FIS score 66) of the 24 controls. The poorest scores were rated in the psychosocial subscale with median 25, compared to zero among controls. At baseline and 18 months after IgRT discontinuation (time point 36 months), the individuals who had to restart IgRT reported a poorer score in all FIS areas compared to those who performed well without IgRT (**Table 2B**). Individuals with IgGsd also reported reduced sexual activity as seen by poorer scores in FIS item 29 (results not shown). In contrast to the SF-36 vitality score, there were no differences in FIS-reported fatigue while on IgRT between the individuals who later had to restart IgRT and those who performed well without it (**Table 2B**). Depression during the study correlated with reported fatigue at baseline (**SI Table 2**). The reported level of fatigue among the study participants at baseline did not correlate with age (results not shown), gender or lung disease (**SI Table 2**). In summary, fatigue is common in IgGsd and most pronounced in those who need IgRT.

**Table 2B:** FIS-score in IgGsd and controls at different time points.

Study subjects	N		FIS physical	FIS cognitive	FIS psychosocial	FIS total score
Baseline						
IgGsd	35	Median	15	14	25	57
		25th-75th percentile	5-24	4-20	12-41	27-83
Controls	24	Median	0	1	0	1
		25th-75th percentile	0-6	0-9	0-12	0-23
			p<0.0001	p<0.0001	p<0.0001	p<0.0001
Need IgRT	18	Median	19	16	38	76
		25th-75th percentile	13-26	11-22	20-41	44-91
No need IgRT	17	Median	13	11	17	37
		25th-75th percentile	4-19	3-17	6-28	14-66
			p=0.02	p=0.03	p=0.02	p=0.04
18 months						
Need IgRT	18	Median	19	16	26	54
		25th-75th percentile	13-26	10-20	17-39	39-83
No need IgRT	16	Median	12	12	19	43
		25th-75th percentile	0-24	0-23	2-43	2-90
			NS	NS	NS	NS
36 months						
Need IgRT	12	Median	20	24	38	82
		25th-75th percentile	11-27	13-26	27-52	66-101
No need IgRT	13	Median	5	5	5	16
		25th-75th percentile	0-15	0-16	0-26	0-57
			p<0.001	p<0.004	p<0.002	p=0.007

FIS, fatigue impact scale; IgGsd, IgG subclass deficiency; IgRT, immunoglobulin replacement therapy; NS, not significant.

Values in bold indicate statistically significant.

### Associations Between SF-36, FIS and EQ-5D-5L VAS

**Figure 3:**
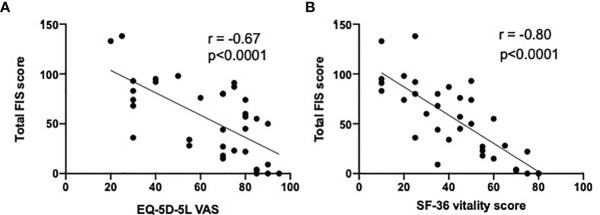
Correlation between EQ-5D-5L VAS score and FIS-total score and SF-36 vitality scores. There was a negative correlation between EQ-5D-5L VAS score and total FIS score **(A)** and SF-36 vitality score **(B)**. r, Spearman correlation coefficient.

The FIS findings were supported by both SF-36 vitality and the EQ-5D-5L VAS scores. There was a strong correlation between total FIS score and SF-36 vitality score as well as between total FIS score and EQ-5D-5L VAS score (**Figure 3A, B**). On a group level, we found no differences in SF-36 vitality scores, or FIS scores at the different time-points, *i.e*. while on IgRT or after discontinuation of the IgRT (data not shown). In summary, there was a good correlation between the different assessment tools.

### Mental Health Issues Are Common in IgG Subclass Deficiency

Mental health issues were more common in IgGsd than in healthy controls, with lower ratings in the mental health dimension of SF-36 (**Table 2A**). The group of individuals who had to restart IgRT were more severely affected with poorer SF-36 mental health ratings, compared to those who performed well without IgRT (**Table 2A**). Six (18%) IgGsd individuals developed depression during the three-year period of this study. One of them had a previous history of depression. Out of the six, three (9%) were diagnosed as having burnout. Five of the patients who developed depression needed to restart IgRT (**SI Table 1**). Depression correlated with FIS total score >60 (**SI Table 2**). Five individuals had a history of depression (two with ongoing anti-depressive treatment at inclusion). These five individuals also reported FIS total scores >60.

### Enrichment of Inflammatory Factors Related to IL-10 Signalling in IgGsd Plasma

To test if systemic inflammation was affected by IgRT and if there was a correlation between systemic inflammation and fatigue in IgGsd, targeted plasma protein profiling was carried out when the participants were on and off IgRT for at least 18 months (baseline or time point 36 months). Of 81 detected proteins, 54 had a predominantly extracellular location and were selected for further investigation. When all IgGsd individuals had been on IgRT for at least 18 months, the plasma levels of 25 factors related to inflammation were dysregulated (**Table 3**). Similar plasma protein patterns were seen in samples collected regardless of whether IgRT was ongoing or not (**Table 3**). The plasma levels of all dysregulated factors, except IL5 and IL17C, were by a good margin above the reported lower limit of detection (**SI Table 3**). The levels of chemokine C-X-C motif ligand (CXCL) 5 and CXCL1, factors which both are important for the activation of neutrophil granulocytes, were the most upregulated factors in IgGsd individuals compared to controls (**Table 3**). The plasma levels of CXCL1 and CXCL5 were estimated to be equal to an analytical level of 10^2^-10^3^pg/mL (Olink Target Inflammation v.95302). We next sought to identify biological pathways relating to the dysregulated plasma protein profiles of IgGsd individuals. Enrichment analysis indicated that IL-10 signalling was the most significantly affected pathway. In total, the nine factors involved in IL-10 signalling were all elevated in the IgGsd group compared to controls (**Table 3**). IL-5 was the only factor that differed significantly when they were on or off IgRT (**SI Figure 2**). Taken together, the plasma protein profiles in IgGsd reflected increased IL-10 signalling and the effect of IgRT on the plasma protein profiles was minimal.

**Table 3:** Dysregulated plasma inflammatory markers in IgGsd.

	HC (n=32)		IgGsd				IgGsd IgRT vs HC	
			IgRT (n=34)		No IgRT (n=29)		Fold change	
Factor	Mean (NPX log2)	SD	Mean (NPX log2)	SD	Mean (NPX log2)	SD	(log2)	P_corr_
**CCL20**	**5.75**	**0.85**	**6.38**	**1.14**	**6.42**	**1.22**	**0.63**	**0.034543**
CCL23	9.58	0.52	9.89	0.50	9.81	0.46	0.31	0.035560
**CCL3**	**3.99**	**0.46**	**4.55**	**0.62**	**4.37**	**0.55**	**0.56**	**0.000769**
**CSF-1**	**9.10**	**0.21**	**9.31**	**0.27**	**9.31**	**0.23**	**0.21**	**0.004514**
**CXCL1**	**6.56**	**1.22**	**8.51**	**0.79**	**8.40**	**1.11**	**1.95**	**<0.000001**
CXCL10	8.85	0.70	9.51	1.19	9.41	1.09	0.66	0.023108
CXCL11	7.33	0.55	8.15	0.94	8.08	0.84	0.82	0.000568
CXCL5	8.07	1.86	10.41	1.26	10.25	1.56	2.34	0.000003
CXCL6	7.34	0.76	8.34	0.87	8.26	0.89	1	0.000066
CXCL9	6.61	0.50	7.42	1.33	7.41	1.12	0.81	0.007327
FGF-23	1.89	0.35	2.18	0.57	2.23	0.52	0.29	0.035942
HGF	8.09	0.31	8.42	0.54	8.35	0.57	0.06	0.011764
IFN-g	5.62	0.78	6.30	1.39	6.40	1.48	0.68	0.040206
IL-17C	0.96	0.38	1.35	0.57	1.20	0.56	0.39	0.006592
**IL10**	**3.08**	**0.37**	**4.14**	**1.12**	**4.03**	**1.08**	**1.06**	**0.000038**
**IL18**	**7.56**	**0.35**	**7.99**	**0.57**	**8.03**	**0.60**	**0.43**	**0.002427**
IL5	-0.32	0.44	1.03	1.33	0.33	1.29	1.35	0.000013
**IL6**	**2.32**	**1.26**	**3.52**	**1.20**	**2.95**	**0.82**	**1.2**	**0.001031**
IL7	1.94	0.71	2.59	0.74	2.62	0.70	0.65	0.002427
**IL8**	**4.07**	**0.72**	**4.65**	**0.81**	**4.43**	**0.81**	**0.58**	**0.010930**
MMP-10	7.71	0.59	8.01	0.91	8.26	0.82	0.3	0.001114
**TNF**	**2.19**	**0.35**	**2.53**	**0.57**	**2.50**	**0.46**	**0.34**	**0.013257**
TNFB	3.75	0.38	4.01	0.51	4.06	0.45	0.26	0.000222
**TNFSF14**	**3.15**	**0.44**	**3.83**	**0.73**	**3.78**	**0.54**	**0.68**	**0.000222**
VEGFA	9.86	0.35	10.18	0.49	10.12	0.49	0.32	0.011334

Factors in bold are associated with IL-10 signalling. NPX, normalized protein expression in Log_2_ scale; HC, healthy controls; IgGsd, immunoglobulin subclass deficiency; IgRT, immunoglobulin replacement therapy; SD, standard deviation; P_corr_, t-test p-value with Benjamin-Hochberg correction for multiple testing.

### Decreased Levels of CSF-1 in IgGsd With Severe Fatigue

**Figure 4:**
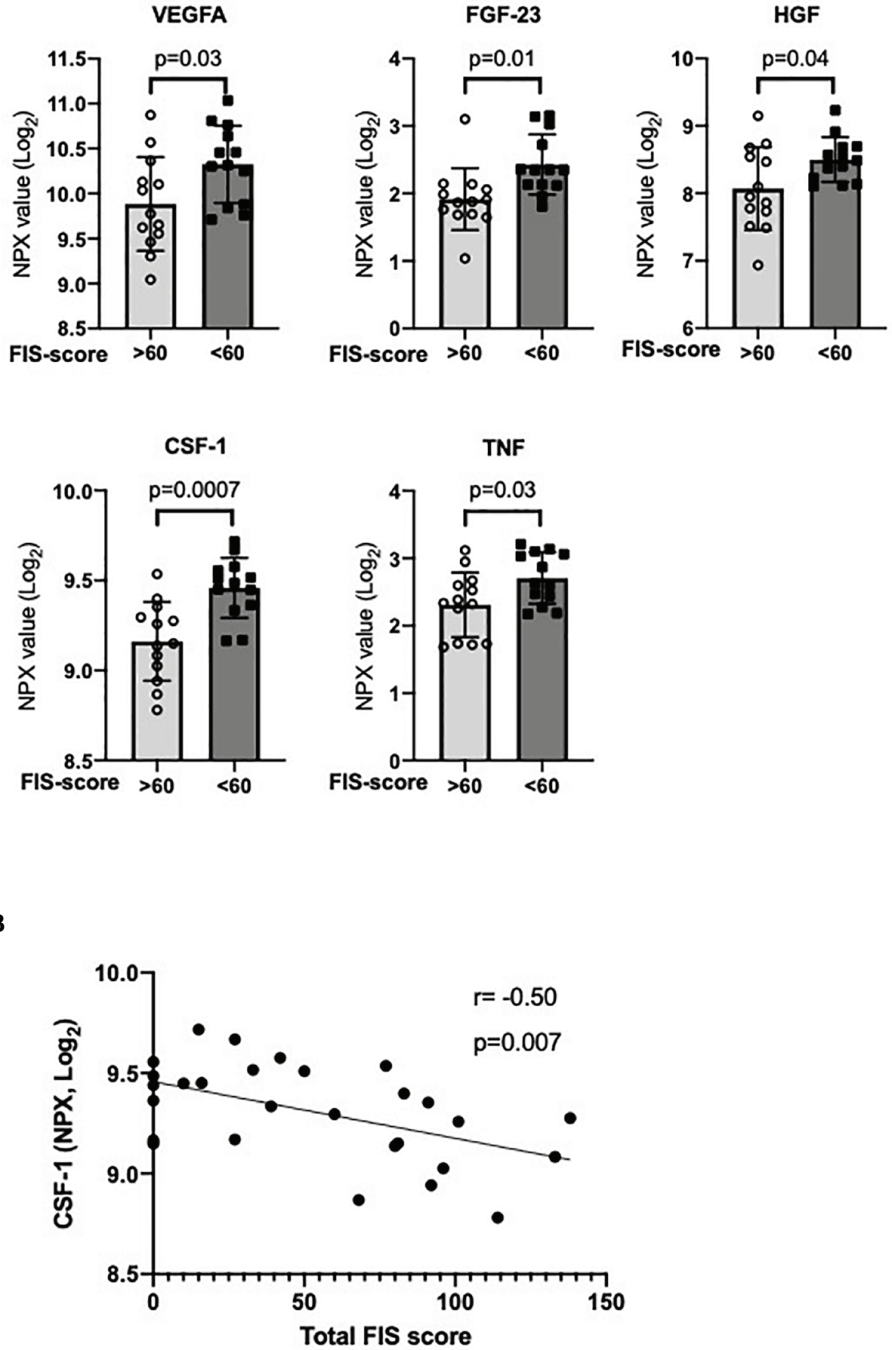
Decreased plasma levels of inflammatory markers in IgGsd with severe fatigue. Plasma levels of the growth factors VEGFA, FGF-23, HGF and CSF-1, and TNF were found in IgGsd with severe fatigue (total FIS score >60, n=13) compared to IgGsd with total FIS score <60 (n=13) when off IgRT **(A)**. Plasma levels of CSF-1 correlated with total FIS score **(B)**. Data is presented as normalized protein expression (NPX) values in Log_2_ scale. Error-bars indicate 95% CI, p represent student’s t-test. VEGF, vascular endothelial growth factor; FGF, fibroblast growth factor; HGF, hepatocyte growth factor; CSF-1, macrophage colony stimulating factor; IgGsd, IgG subclass deficiency; FIS, fatigue impact scale; IgRT, immunoglobulin replacement therapy; r, Spearman correlation coefficient.

Next, we used the FIS total score to investigate the relationship between severe fatigue and circulating inflammatory markers. Plasma protein profiles were compared between IgGsd individuals with a total FIS score above or below 60, when off IgRT. Plasma levels of CSF-1, vascular endothelial growth factor (VEGF-A), fibroblast growth factor (FGF-23), hepatocyte growth factor (HGF) and TNF were lower in individuals with severe fatigue, *i.e.* FIS>60 (**Figure 4A**). CSF-1 also showed a moderate correlation with total FIS score in IgGsd, when off IgRT (**Figure 4B**). These findings indicate that there may be an association between fatigue and the inflammatory response in IgGsd.

## Discussion

This longitudinal prospective study is the first to analyse health-related QoL and fatigue in individuals with IgGsd while on and after discontinuation of IgRT, and it also includes plasma profiling of inflammatory factors. Lower health-related QoL and a higher prevalence of fatigue were reported among IgGsd individuals compared to healthy controls. Furthermore, 18 (53%) of a total of 34 individuals who completed the study needed to restart IgRT. QoL and fatigue correlated with the need to reintroduce treatment. IgGsd was associated with signs of systemic inflammation and increased IL-10 signalling, regardless of whether IgRT was ongoing or not. Notably, severe fatigue was associated with reduced plasma levels of the microglial stimulating factor CSF-1 and several other growth factors.

The current study indicated reduced QoL in all dimensions of the SF-36 questionnaire for IgGsd individuals, with the poorest scores for general health, and vitality, regardless of whether IgRT was ongoing or not. The self-rated QoL in IgGsd displayed similarities to an Italian cohort of CVID and to an Iranian cohort of PADs, where the poorest scores also were reported in general health (10, 30).

The EQ-5D-5L VAS scores reported by the IgGsd group were comparable to what has been reported by two large Swedish study cohorts of Multiple Sclerosis (median: 75; inter quartile range: 60-88) and heart failure (mean: 63; standard deviation: 20), both conditions where fatigue is an important comorbidity (31, 32). The EQ-5D-5L VAS score reflects the respondents’ overall assessment of QoL and detects clinically significant changes. We found a strong correlation between the EQ-5D-5L VAS score and FIS. Therefore, EQ-5D-5L VAS, which is both fast and easy to use, appears to be of benefit when screening individuals with IgGsd for fatigue.

Fatigue is an important variable in perceived health, but assessment is complex due to its multidimensional character. The SF-36 dimension of vitality is a general measure of fatigue, and the poor vitality scores indicated that fatigue had a significant impact on QoL in individuals with IgGsd. However, any chronic condition can negatively affect the vitality score (33). A significant impact of fatigue on QoL in CVID and other PADs using generic questionnaires has been reported (8, 12, 34). In a cohort of paediatric patients with primary immunodeficiency nearly 20% reported severe fatigue when assessed with a paediatric fatigue scale. Perceived fatigue was not related to ongoing infections (35). Assessment of fatigue by the FIS questionnaire revealed poorer scores in all assessed areas in the IgGsd group compared to controls. The reported levels of fatigue were independent of the age, comorbidity, and sex of the study participants. Moreover, IgGsd scores were even poorer in the cognitive and social dimensions than in Multiple Sclerosis (36). The poorest FIS scores were among those IgGsd individuals who needed to reintroduce the IgRT, suggesting that observed fatigue is associated with increased susceptibility to bacterial infections. The prevalence of depression in our cohort was 18%, which is slightly lower than 23-25% reported in cohorts of heterogeneous PADs (11, 37), but higher than the prevalence (8%) of depression in the general Swedish population (38). Our results are in line with a recent report among individuals with PADs in which vitality was the most affected dimension related to QoL (39). In CVID, fatigue has been attributed to a “wear off” effect in individuals subjected to intravenously administrated IgRT (8, 11). However, a “wear off” effect is not likely to have been a factor in our study since there were no significant differences at a group level in FIS score when on and off IgRT. Additionally, Ig was administrated subcutaneously to all individuals. The FIS assessment considers several more aspects of fatigue compared to SF-36 vitality and can thus be considered more comprehensive. For instance, FIS revealed a negative impact on sexual activity in the IgGsd group. The negative consequences of living with PADs on sexual relations have to our knowledge not previously been reported. Overall, total FIS scores correlated strongly to SF-36 vitality scores, as well as the EQ-5D-5L VAS scores. In summary, our results show that fatigue is a common problem in IgGsd and negatively affects QoL. Although individuals that needed to reintroduce IgRT seemed to suffer from more severe fatigue, IgRT did not have any significant impact on perceived fatigue. Those who needed to restart IgRT had poorer FIS scores already at baseline.

Fatigue is part of the sickness behaviour induced by the inflammatory reaction (17–19). The relationship between fatigue and the level of inflammation is unclear (40, 41). IL-5 was the only factor directly elevated by IgRT. IL-5 is an important driver of T helper (h) 2 deviation and reduced levels when not on IgRT may indicate increased Th1 polarisation. Th1 deviation and increased plasma levels of interferon-gamma have previously been reported in a group of patients with CVID with poor prognosis (42). The IgGsd plasma protein profiles were characterised by increased levels of the neutrophil activating chemokines CXCL1 and CXCL5, and enrichment of dysregulated factors related to IL-10 signalling, suggesting that the underlying pathophysiology of IgGsd is different compared to CVID. IL-10 is an immune regulatory factor important for the homeostatic control of infection and inflammation (43). Pathway analysis revealed enrichment of pro-inflammatory factors (i.e. CXCL1, IL-8, TNF, CSF-1 and CCL3) associated with IL-10 signalling, which were all more abundant in the IgGsd group than in the healthy controls. Thus, increased IL-10 signalling in IgGsd is likely to be secondary to an underlying inflammatory response.

Serum signatures dominated by pro-inflammatory cytokines have also been reported to correlate with CFS/ME disease severity, supporting a link between systemic inflammation and fatigue (44). We found decreased levels of CSF-1 in IgGsd complicated by severe fatigue. The receptor of CSF-1 is expressed by microglial cells in the brain, and studies indicate that CSF-1 prevents inappropriate microglial activation at steady-state (45). Mutations in the CSF-1 receptor induce a rare form of early-onset dementia (46). Treatment with recombinant CSF-1 attenuated neuroinflammation in an experimental study on hypoxic brain injury (47). CSF-1 deficient mice develop behavioural defects (48). Together these findings imply that there may also be a link between reduced levels of CSF-1 and fatigue, and it would be beneficial to include research on cerebrospinal fluid in future studies.

In addition, decreased plasma levels of several growth factors were related to severe fatigue in IgGsd. HGF and VEGF are neurotrophic factors (49, 50) and decreased expression of HGF has been reported in depression (51).

Altered expression levels of neurotrophic factors may contribute to abnormal neuroplasticity and psychopathological disorders such as fatigue (50). FGF-23 acts in an endocrine manner and contributes to the regulation of vitamin D synthesis (52). Vitamin D signalling is proposed to be a potent regulator of immunity (53). Low levels of vitamin D are associated with increased susceptibility to infections (54) and neuropsychiatric disease (55). Thus, decreased levels of FGF-23 may contribute to fatigue by several mechanisms. To summarise, an imbalance between pro-inflammatory factors and trophic factors may contribute to severe fatigue in IgGsd.

The prospective design and the use of the same cohort on and off IgRT for evaluation of health-related QoL and fatigue are strengths of this study. Healthy controls who did not suffer from chronic inflammatory diseases were matched for age and sex. The SF-36 and EQ-5D-5L VAS instruments are well-established and have been validated in many diagnoses. SF-36 has also been validated for PADs. Overall, the consistent results between the different instruments used in the study strengthen our findings.

The restricted number of study subjects is a major limitation of the study, and the findings need to be verified in other, larger cohorts of individuals with IgGsd. The use of a disease-specific QoL questionnaire would have added value (56). FIS has been developed to assess fatigue in multiple sclerosis and is not validated for PADs. It has however, been extensively used in other chronic diseases (57). The study shows a strong correlation between total FIS score and SF-36-vitality, which suggests that FIS can be used for comprehensive assessment of fatigue in PADs. Plasma protein profiling with PEA technology only provides a relative quantification of the proteins present in the samples but is a valuable tool for exploratory studies. However, candidate markers such as CSF-1 and IL10 need to be validated in quantitative assays.

In conclusion, this prospective longitudinal study demonstrates that fatigue is a major contributory factor to impaired health-related QoL in IgGsd. Severe fatigue in IgGsd was associated with decreased expression of neurotrophic growth factors and correlated with decreased plasma levels of CSF-1. Perceived fatigue was most pronounced among IgGsd individuals needing IgRT to alleviate the burden of infection. Our conclusion is therefore that severe fatigue seems to be a marker for the need of continuous IgRT. Currently, the decision on whether to introduce IgRT or not, in a treatment-naïve patient, is based on the severity and burden of infections. We suggest that, in addition to this, the individual’s degree of fatigue should be thoroughly assessed, and taken into account.

## Data Availability Statement

The raw data supporting the conclusions of this article will be made available by the authors, without undue reservation.

## Ethics Statement

The studies involving human participants were reviewed and approved by The Ethical Review Board in Linköping, Sweden (Dnr 2011/506-31). The patients/participants provided their written informed consent to participate in this study.

## Author Contributions

Material preparation, data collection and analysis were performed by PW, ÅN-A, MN, JB, CD and SN. The manuscript was written by PW, CD and SN. ÅN-A, MN and JB edited the manuscript. All authors have contributed, read, and approved the submitted manuscript. All authors agree to be responsible for all aspects of the work.

## Funding

The study has been funded by grants from FORSS and Futurum, both institutions promoting research in the County of Jönköping, and ALF grants Region Östergötland.

## Conflict of Interest

PW and ÅN-A are members and JB is a former chair of the Swedish National Guideline Committee for primary immune deficiencies.

The remaining authors declare that the research was conducted in the absence of any commercial or financial relationships that could be construed as a potential conflict of interest.

## Publisher’s Note

All claims expressed in this article are solely those of the authors and do not necessarily represent those of their affiliated organizations, or those of the publisher, the editors and the reviewers. Any product that may be evaluated in this article, or claim that may be made by its manufacturer, is not guaranteed or endorsed by the publisher.
