# Supplementary material for: Fatigue Is Common in Immunoglobulin G Subclass Deficiency and Correlates With Inflammatory Response and Need for Immunoglobulin Replacement Therapy
Source: Front Immunol. 2022 Jan 10;12:797336. doi: 10.3389/fimmu.2021.797336 (PMC8785394; doi:10.3389/fimmu.2021.797336)
Supplement: Supplementary file 1 [file DataSheet_1.pdf]

Fatigue is common in immunoglobulin G-subclass deficiency and correlates with inflammatory response and need of immunoglobulin replacement therapy

Per Wågström[1], Åsa Nilsdotter-Augustinsson[2], Mats Nilsson[3], Janne Björkander[4], Charlotte Dahle[5], Sofia Nyström[5,6]

1. Department of Infectious Diseases, Ryhov County Hospital, Jönköping and Department of Biomedical and Clinical Sciences, Linköping University, Linköping, Sweden

2. Department of Infectious Diseases and Department of Biomedical and Clinical Sciences, Linköping University, Linköping, Sweden

3. Futurum, Region Jönköping County, and Department of Health, Medicine and Caring, Linköping University, Linköping, Sweden

4. Division of Clinical Immunology, Department of Biomedical and Clinical Sciences, Linköping University, Linköping, and Wetterhälsan Health Care Center, Jönköping, Sweden.

5. Department of Clinical Immunology and Transfusion Medicine and Department of Biomedical and Clinical Sciences, Linköping University, Linköping, Sweden

6. Division of Molecular Medicine and Virology, Department of Biomedicine and Clinical Sciences, Linköping University, Linköping, Sweden

Supplementary figures and tables

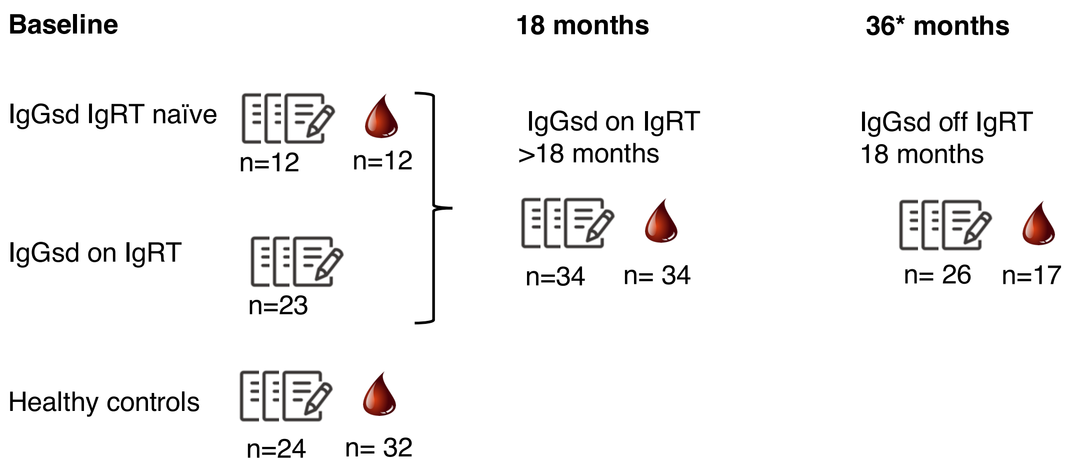

SI Figure 1. Overview of study protocol. Evaluation of quality of life (QoL) and blood sampling were performed at three occasions in individuals with IgGsd: At baseline (study month 0), after 18 months on IgRT and at 18 months after discontinuation of IgRT (time point 36 months). Healthy controls were correspondingly evaluated and sampled on one occasion. *Base-line blood samples were analysed from IgRT naïve individuals, instead of discontinuation samples collected at 36 months. IgGsd, IgG subclass deficiency, IgRT, Ig-replacement therapy.

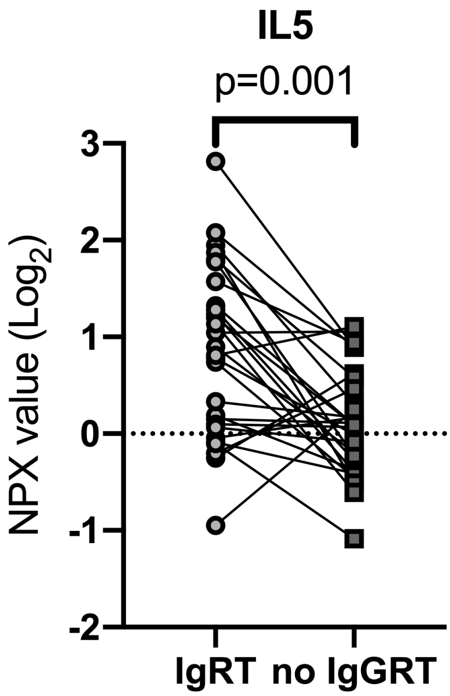

SI Figure 2. IgRT was associated with increased plasma levels of IL-5 in IgGsd. Data is presented as normalized protein expression (NPX) values in Log_2 scale. Error-bars indicate 95% CI, p, paired t-test.

SI Table 1. IgGsd subsets and the need for IgRT

	Total	Need for IgRT	No need for IgRT	
All IgGsd, n (%)	34#	18 (51)	16 (49)	
IgGsd subset, n (%)				*p-value*
Females, n (%)	21	11 (52)	10 (48)	0.29
Lung disease	16	11 (69)	5 (31)	0.33
Depression*	6	5 (83)	1(17)	0.18

#One participant left the study. *Depression during the study period. Fisher's exact test was used.

SI Table 2. IgGsd subsets and severe fatigue

	Total	FIS>60	FIS≤60	
All IgGsd, n (%)	34[#]	19 (56)	15 (44)	
IgGsd subset, n (%)				*p-value*
Females, n (%)	21	12 (57)	9 (43)	0.99
Lung disease	16	10 (63)	6 (36)	0.51
Depression*	6	6 (100)	0 (0)	0.02

[#]One participant left the study. *Depression during the study period. Fisher's exact test was used.

SI Table 3. Detection levels and linearity of Olink Target Inflammation panel*

Factor	Range in study population, NPX (log2)	LLOD NPX(Log2)	LLOD pg/mL	Linearity pg/mL
CCL20	4.0-9.5	0.85	7.63	7.6-15625
CCL23	8.1-11.4	0.27	30.5	30.5-31250
CCL3	2.8-5.9	-0.69	0.06	0.06-488
CSF-1	8.7-10.1	-0.32	0	0.01-1953
CXCL1	4.5-10.2	0.54	3.81	7.6-15625
CXCL10	6.7-12.1	1.32	7.63	7.6-15625
CXCL11	5.9-10.1	0.36	7.63	30.5-15625
CXCL5	5.2-12.5	0.96	0.95	0.95-7812
CXCL6	6.3-10.5	-0.24	7.63	30.5-15625
CXCL9	4.4-10.6	0.38	0.95	0.95-3906
FGF-23	0.96-3.93	0.85	122	122-62500
HGF	6.9-9.5	0.10	7.63	7.63-125000
IFN-g	3.7-10.2	1.79	0.24	0.24-15625
IL-17C	0.14-3.0	0.79	30.5	30.5-125000
IL10	1.4-7.7	0.74	0.48	0.48-62500
IL18	6.6-9.9	0.86	3.81	0.06-15625
IL5	-1.2-6.9	0.23	3.81	3.81-15625
IL6	0.86-8.4	0.62	0.12	0.12-3906
IL7	0.75-4.1	0.21	0.24	0.24-7812
IL8	2.6-6.6	-0.33	0.03	0.03-3906
MMP-10	6.7-11.1	0.64	0.95	0.95-15625
TNF	1.6-4.0	-0.73	0.95	0.95-31250
TNFB	3.0-5.3	0.27	0.24	0.48-15625
TNFSF14	2.4-5.9	0.11	0.95	1.91-15625
VEGFA	8.9-11.4	-0.41	0.06	0.06-7812

*Validation data reported by Olink, based on samples spiked with recombinant proteins.
